# Nondestructive Examination of Carbon Fiber-Reinforced Composites Using the Eddy Current Method

**DOI:** 10.3390/ma16020506

**Published:** 2023-01-04

**Authors:** Ryszard Łukaszuk, Tomasz Chady

**Affiliations:** 1 Doctoral School, West Pomeranian University of Technology, 70-313 Szczecin, Poland; 2 Faculty of Electrical Engineering, West Pomeranian University of Technology, Sikorsky 37 St., 70-313 Szczecin, Poland

**Keywords:** nondestructive testing (NDT), nondestructive evaluation (NDE), eddy current testing (ECT), carbon fiber-reinforced composites, differential eddy current probe, hidden flaws detection

## Abstract

This paper presents the results of experiments using the eddy current system designated for nondestructive inspection of carbon fiber-reinforced composites. For this purpose, the eddy current testing system with a differential transducer with two pairs of excitation coils oriented perpendicularly and a central pick-up coil was utilized. The transducer measures the magnetic flux difference flowing through the pick-up coil. The transducer of this design has already been successfully utilized to inspect isotropic metal structures. However, the anisotropy of the composites and their lower conductivity compared to metal components made the transducer parameters adjustment essential. Thus, various excitation frequencies were considered and investigated. The system was evaluated using a sample made of orthogonally woven carbon fiber-reinforced composites with two artificial flaws (the notches with a maximum relative depth of 30% and 70%, respectively, thickness of 0.4 mm, and a length of 5 mm). The main goal was to find a configuration suitable for detecting hidden flaws in such materials.

## 1. Introduction

Composites are manufactured by joining together two materials that differ significantly in their chemical and physical properties. The undoubted advantages of composites are simple modifiability of the structure for the target use, low production cost, good corrosion resistance, and high strength–thickness ratio. Due to these facts, composites found extensive use in various branches of modern industry, such as shipbuilding (machine enclosures) [1], offshore (pipelines) [2], civil engineering (reinforced foundations, sewage pipes) [3], power engineering (rotor blades in wind turbines) [4], aerospace (crew capsules, satellites) [5], and even in biomedicine (implants) [5]. Composites are the first choice in case of weight reduction, fire and thermal protection, and invariable dimensions or stiffness. Unfortunately, the strength of composites, like the steel commonly used in industry for years, is limited. Moreover, the composite structure damage may already occur at the manufacturing stage and substantially affect the structure’s performance and lifetime. For example, in the power energy industry, the production of larger wind turbine blades causes an increased failure rate due to the weight reduction of the glass composite [6,7]. Carrol et al. indicate that wind turbine blade malfunctions account for 6.2% of failure cases [8]. Sometimes, a cost-intensive structural repair is necessary to resume the turbine operation. Health monitoring and frequent inspections, especially nondestructive ones, are necessary to enhance the productiveness of the turbines [9]. In the automotive industry, the production of hydrogen fuel cell vehicles is gaining popularity, given the need to reduce fossil fuel consumption and air pollution [10]. Hydrogen storage vessels are manufactured from carbon fiber-reinforced composites, which are lightweight and corrosion-resistant compared to those made of steel. To ensure structural integrity and thus a safe and reliable operation of the composite vessel, the detection and identification of structural defects by using nondestructive techniques are invaluable [11].

Exemplary defects in composites include fiber breakages, cracks, porosities, fiber misalignments, and delaminations [1,2]. Therefore, it is essential to perform nondestructive testing of composites at the production stage and during the operation. Systematic inspections increase the safety of structure service and diminish the danger to the life or health of the maintenance and end users.

Nowadays, in the case of composites, the following techniques of nondestructive testing are utilized: the ultrasonic method, eddy currents, thermography, radiography, and shearography. The eddy current method is widely used in the nondestructive evaluation of metal components. This method is advantageous due to surface and subsurface defect detection, the possibility of coating thickness measurement, and a non-hazardous, contactless procedure [12,13]. An important parameter is the penetration depth of eddy currents, which depend on the choice of the excitation frequency. The eddy current method is promising for all composites consisting of materials with good electrical conductivity, such as those reinforced with carbon fiber. However, it requires further study to optimize the design of the transducers and the testing parameters following the carbon fiber-reinforced composite specificity. It is predominately due to the relatively low conductivity and material anisotropy [1].

Moreover, carbon fiber-reinforced composites differ in reinforcement type and fiber alignments. Components with uni-, bi-, or multi-directional fiber orientations are symmetrically built. However, there are also many components with reinforcement made of orthogonally woven fibers. Nondestructive inspection of composites with such a fiber alignment is complex due to asymmetry and high anisotropy. Therefore, it is especially challenging to detect hidden flaws in such materials.

## 2. Materials and Methods

Several studies were already performed concerning the alignment of the eddy current method to composite specificity. For example, Mizukami et al. proposed a new approach to identify and localize delamination in carbon fiber-reinforced composites. The study involved the inspection of quadratic samples (200 mm × 200 mm) with artificial delamination produced using a thin polyimide film placed between composite layers at the lamination stage [14]. Dehui et al. focused on the detection of crack detection (inner flaws with dimensions of around 10 mm × 0.2 mm × 0.4 mm) using a new method relying on power loss measurements. Orthogonally woven samples and samples with uni-, bi-, and four-directional carbon fibers and dimensions of around 200 mm × 200 mm were inspected [15]. Pasadas et al. proposed an approach based on guided wave tomography and eddy currents to detect and localize fiber breakages. A rectangular sample (500 mm × 470 mm) with four-directional fibers (0°, 90°, 45°, −45°) containing an artificially made breakage with a length of 20 mm was subjected to experiments [16]. The Fraunhofer Institute for Nondestructive Testing researched the application of the high-frequency eddy current method to carbon composites. The research involved the detection of hidden defects such as web faults and delaminations [17]. Zeng et al. proposed the eddy current method to detect fiber waviness in carbon composites using a transducer with vertical coils. The research comprised a rectangular, unidirectional sample (200 mm × 200 mm) [18]. Fan et al. used a differential rectangular sensor to detect delaminations, cracks, and impact damages using a technique based on pulsed eddy current [19]. Cheng et al. detected wrinkles, missing bundles, and gaps in carbon fiber-reinforced composites using a high-resolution, self-nulling eddy current transducer [20]. The research conducted by Underhill et al. emphasized the nondestructive evaluation of sandwich panels made of carbon composites. The examination procedure involved an eddy current array sensitive to minor disbound and dents [21].

This study used a rectangular sample (labeled S03) made of an orthogonally woven carbon fiber-reinforced composite as the subject of the experiments and analysis. An overview of the sample parameters is provided in Table 1.

**Figure 1:**
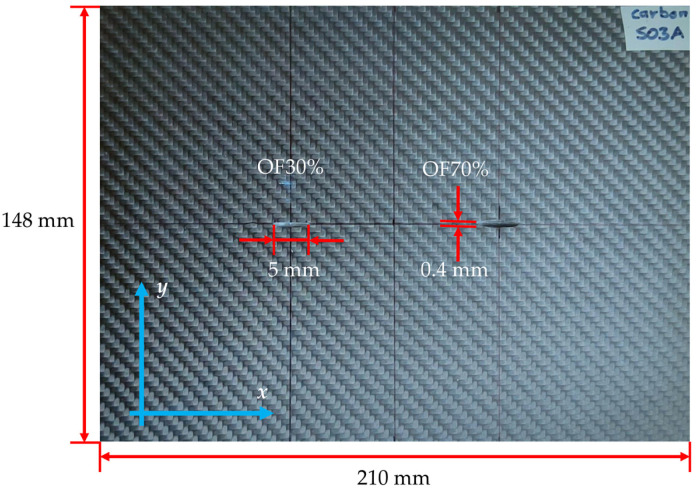
Photo of the sample S03 with the two artificial flaws (**left**: OF30%, **right**: OF70%). The transducer was scanned over the opposite side of the plate.

The sample contained two artificial flaws (cuts) manufactured with a diamond blade. Two semi elliptical cuts with a thickness of 0.4 mm and a length of 5 mm were made on the longitudinal axis of the plate symmetry. The relative maximum depth of the cuts is 30% (OF30%; OF stands for Outer Flaw) and 70% (OF70%) of the material thickness, respectively. The outer flaw means that the transducer was scanned over the opposite side of the specimen. The photo of the sample is shown in Figure 1.

The eddy current method is a nondestructive technique applied to inspect conductive materials. An alternating current flowing through the excitation coil creates a primary alternating electromagnetic field. The field covers the sample and causes the eddy current to flow into it. If an inhomogeneity occurs in the material, the current path is disrupted. Eddy currents induce a secondary magnetic field that causes the current flow in the field-sensing coil. Measurement and analysis of the field-sensing coil voltage enable the assessment of the material’s condition [22].

**Figure 2:**
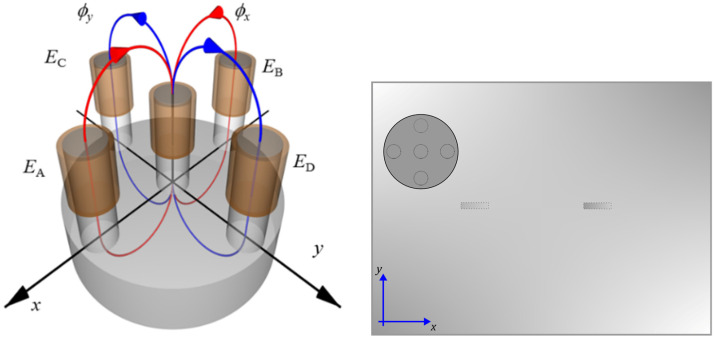
Schematic view of the differential eddy current transducer and arrangement over the sample. *E_A_*, *E_B_*, *E_C_*, *E_D_*—excitation coils, *S*—pick-up coil, *core*—ferrite core, *φ_x_*, *φ_y_*—magnetic fluxes generated by the pairs of excitation coils.

**Figure 3:**
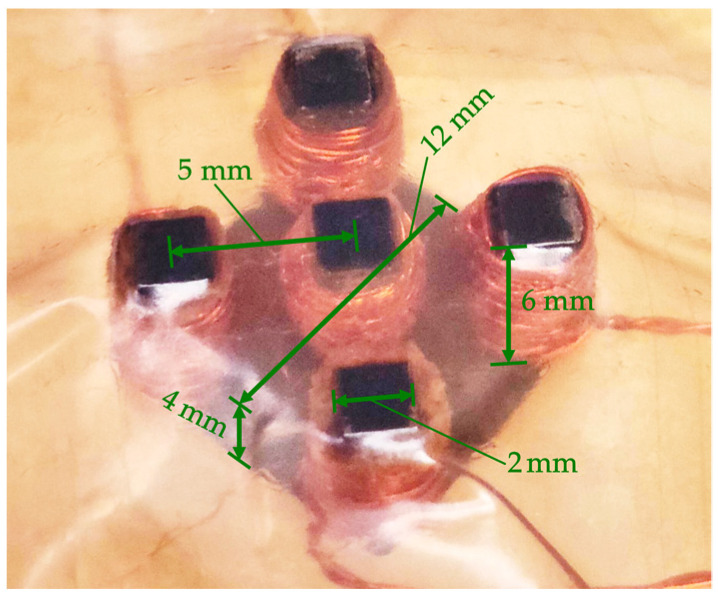
Photo of the transducer protected by the tape (bottom view).

A differential eddy current transducer presented in Figure 2 and Figure 3 was utilized for testing. The transducer design was proposed and successfully used by Chady et al. [23] for testing isotropic, metal-made Inconel structures. The transducer comprises a ferrite core with five columns (Figure 2). The columns are symmetrically arranged. The middle column carries a pick-up coil *S*, while the other four carry excitation coils *E_A_*, *E_B_*, *E_C_*, and *E_D_*. The excitation coils form two pairs arranged perpendicularly. If the test material is homogeneous, the magnetic fluxes between the coils *E_A_* and *E_B_* and between the coils *E_C_* and *E_D_* are almost the same. The inaccuracies in the transducer implementation cause minor differences between the fluxes. The fluxes are forced in opposite directions. Therefore, the voltage induced in the pick-up coil *S* is nearly zero. The heterogeneity of the material structure caused by defects results in larger flux differences and the increased voltage induced in coil *S.* Table 2 presents chosen parameters of the transducer.

**Figure 4:**
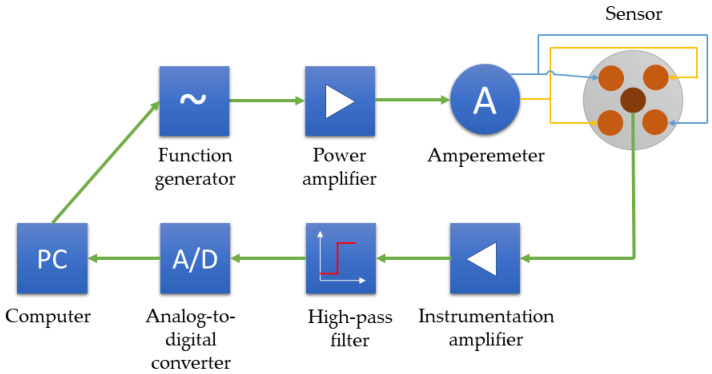
Measuring system.

Figure 4 shows a simplified block scheme of the measuring system. The system comprises the eddy current differential transducer (Figure 3), a function generator, amplifiers, an analog-to-digital converter, an amperemeter, a high-pass filter, and a control computer. First, the computer sends the parameters of the requested excitation (amplitude, frequency, gain) to the function generator, which produces an analog excitation voltage on this basis. Then, the power amplifier boosts the voltage signal and passes it to the excitation coils of the transducer. The amperemeter between the amplifier and the transducer enables observation of the excitation current. Subsequently, the instrumentation amplifier boosts the output voltage from the transducer, and the Butterworth fourth-order high-pass filter reduces lower-frequency interferences. Eventually, the converter transforms the output voltage from its analog form into digital, which is saved in the computer for further analysis.

## 3. Results of Experiments and Discussion

### 3.1. Preliminary Investigation of the Transducer

Before the main experiments, a preliminary evaluation of the transducer performance was carried out. For this purpose, the voltage from the pick-up coil was measured in two cases:

(a) the transducer was placed over the central unflawed part of the sample, and the voltage *U*_1_ was measured
(b) the transducer was placed at the edge of the sample (one of the excitation coils was outside the sample), and the voltage *U*_2_ was measured.

The measurements were repeated for frequencies starting from 500 kHz up to 4.5 MHz (with the step of 50 kHz). For each frequency, the voltage *U*_1_ measured in case a) and voltage *U*_2_ measured in case b) were subtracted from each other and the relative voltage changes were calculated using the formula (1):

(1) $$\delta u=\frac{\left|U_{2}-U_{1}\right|}{U_{1}}$$

**Figure 5:**
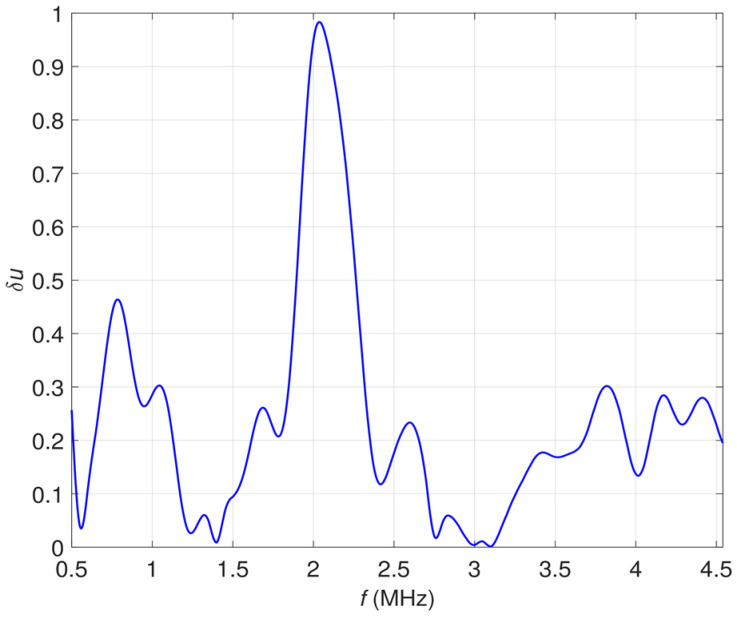
Relative voltage changes *δu* caused by the material influence plotted as a function of the excitation frequency *f*.

All the resulting values were utilized to create the frequency characteristic of the transducer’s sensitivity shown in Figure 5.

The achieved characteristic was used to select the excitation frequency range for further experiments. As the maximum changes of the signal were observed around a resonance frequency (*f* = 2 MHz), the subsequent investigations were conducted for the frequencies starting from 1 MHz up to 2 MHz.

### 3.2. Measurement and Data Processing

The final experiments consisted of 1D multifrequency testing (first stage) and 2D single-frequency inspection (second stage). The sample was placed under the differential eddy current transducer fixed to the head of the positioning system. The transducer was moved along the *x*-axis (Figure 1) on the sample’s surface opposite the defects. After applying the excitation of the selected frequency, the transducer was moved over the sample. The voltage from the pick-up coil was measured every 0.5 mm and stored in the computer for further analysis. The inspection was repeated for different frequencies. In the second stage, the measurement was carried out for a single frequency of 2 MHz. The frequency was selected based on the results achieved for 1D scanning. Analogically to the first stage, the transducer was moved along the *x*-axis, and the pick-up coil voltage was measured every 0.5 mm. Subsequently, the procedure was performed for successive transducer positions (every 1 mm) along the *y*-axis (Figure 1).

In order to simplify the comparison of the results of measurements, the relative voltage difference was calculated using the formula (2):

(2) $$∆u=\frac{∆U}{∆U_{max}}$$

where: ∆U—voltage difference (voltage changes concerning the voltage measured for the defect-free part of the sample) calculated for a given frequency, $∆U_{max}$—maximum of voltage changes measured for all frequencies.

### 3.3. Results of Multifrequency 1D Examination

**Figure 6:**
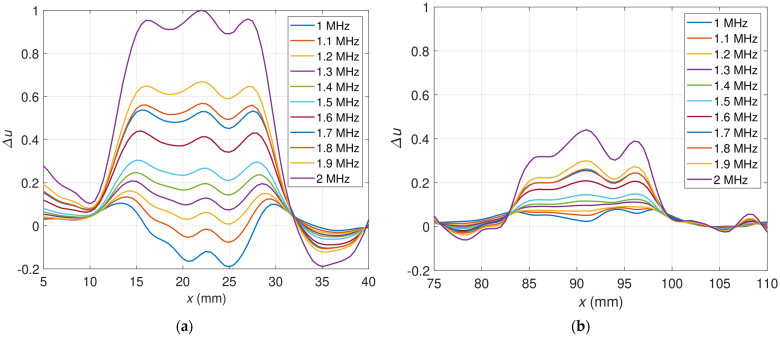
Relative voltages for various excitation frequencies as a function of sensor position (**a**) OF70%; (**b**) OF30%.

Figure 6 presents a set of curves representing relative voltage differences ∆u for distinct frequencies as a function of transducer position along the *x*-axis. The signals were plotted separately for the flaw OF70% and the flaw OF30% due to the significantly different values. As shown in Figure 6a for the flaw OF70%, the curves for the frequencies 1 MHz–1.6 MHz are quasiconvex and increase their values along with the frequency rise. It is noticeable that the extreme signal values corresponding to the flaw location are negative for frequencies between 1 MHz and 1.1 MHz. For frequencies above 1.1 MHz, the extreme signal value around the flaw is positive. Starting from the frequency 1.7 MHz, the shape of the curve changes to quasiconcave and continues to grow the peak value. Figure 6b presents the signals measured for the flaw OF30%. One can observe that curves representing relative voltage as a function of transducer position differ from those obtained for the flaw OF70%. Peak values of the signals are positive for all the excitation frequencies. The transition from the quasiconvex to the quasiconcave curve shape occurs for a frequency of 1.3 MHz. Similarly to the signals for the flaw OF70%, the peak values increase with the rise of the excitation frequency.

**Figure 7:**
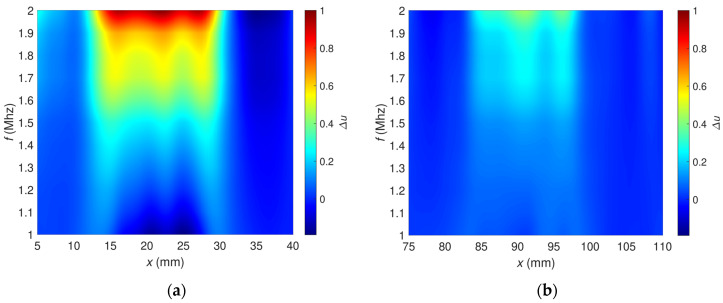
Spectrograms of relative voltage (**a**) OF70%, (**b**) OF30%.

The signals obtained for different frequencies can be presented in a single plot called a spectrogram. In this case, the abscissa corresponds to the transducer position *x*, the ordinate corresponds to the frequency *f*, and the colors represent the value of the signal. Figure 7 shows the spectrograms for both artificial flaws. The flaw OF70% (Figure 7a) is readily detectable across the entire frequency spectrum. A different situation occurs regarding the flaw OF30% (Figure 7b). The flaw is barely visible for the frequency values from 1 MHz up to 1.5 MHz. Its visibility improves for the higher frequencies. Both flaws are most detectable for the frequency of 2 MHz, which is close to the natural resonance frequency of the transducer circuit.

The resonance phenomenon caused a disturbance in the shape of the frequency characteristics of defects that can be observed in the case of metal samples [23]. The range of excitation frequencies used for metals (1 kHz–200 kHz) did not include the inherent resonant frequencies of the measuring transducer. In such a case, the shape of the characteristic depends on the penetration depth for different frequencies and the defect’s depth. Using resonant frequencies for measurements made it possible to obtain a greater sensitivity of the transducer to defects, but on the other hand, it made identifying defects more difficult.

**Figure 8:**
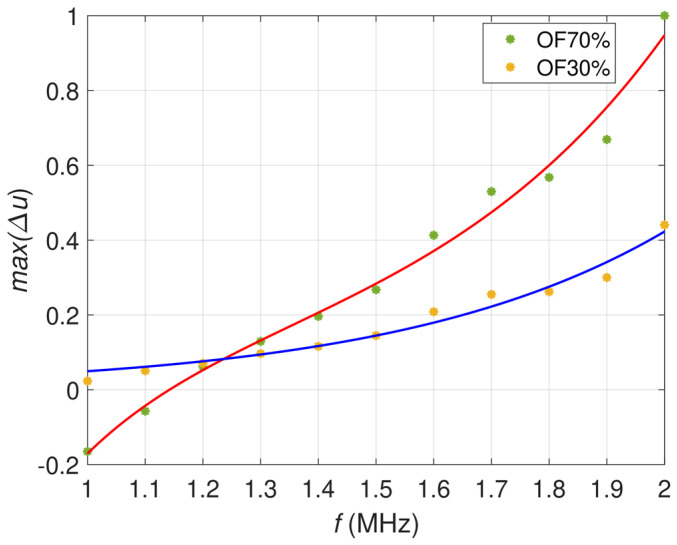
Maximum relative voltages as a function of the excitation frequency. The red line approximates data for OF70%, while the blue line approximates data for OF30%.

Figure 8 comprises curves of the peak value of relative voltage changes ∆u as a function of the excitation frequency *f*. It is evident that for the frequencies of 1 MHz–1.2 MHz, the curve corresponding to the OF70% takes negative values. The curves intersect between frequencies 1.2 MHz and 1.3 MHz. The transition from the positive to negative values for OF70% occurs because the transducer works with two perpendicular coil pairs whose signals balance each other. Based on the analysis of the curves, the location of defects can be quickly and unambiguously determined, for example, using the frequency for which the curve crosses the *f*-axis. For the flaw OF70%, this frequency is 1.15 MHz; for OF30%, the intersection point does not exist in the range of measured values.

### 3.4. Results of Two-Dimensional Examination with a Single-Frequency Excitation

**Figure 9:**
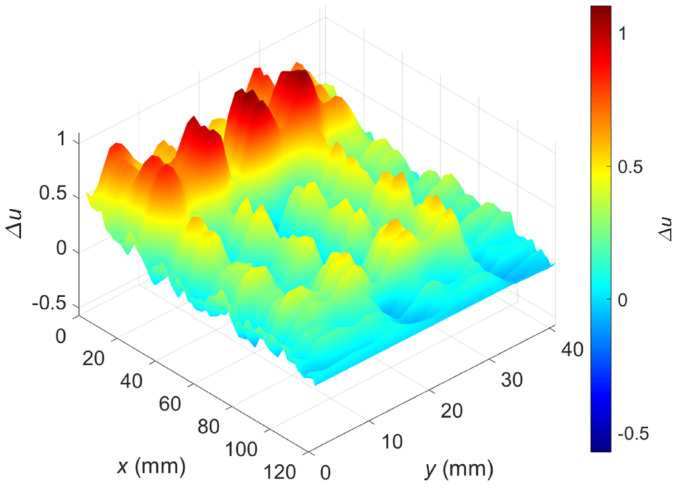
Two-dimensional plot of the relative voltage as a function of the sensor position; excitation frequency *f* = 2 MHz; a raw signal before background removal.

**Figure 10:**
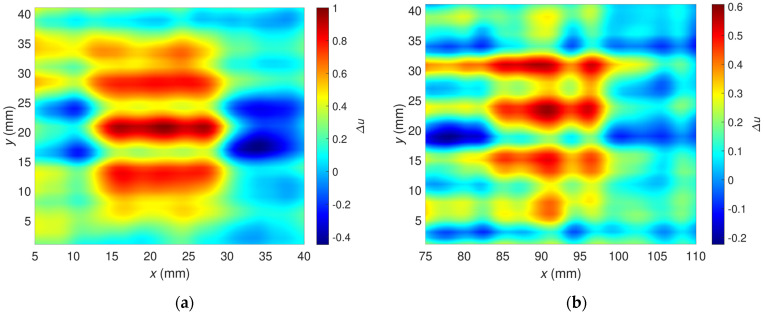
Two-dimensional plots of the relative voltage as a function of sensor position; excitation frequency *f* = 2 MHz; the raw signals before background removal; (**a**) OF70%, (**b**) OF30%.

**Figure 11:**
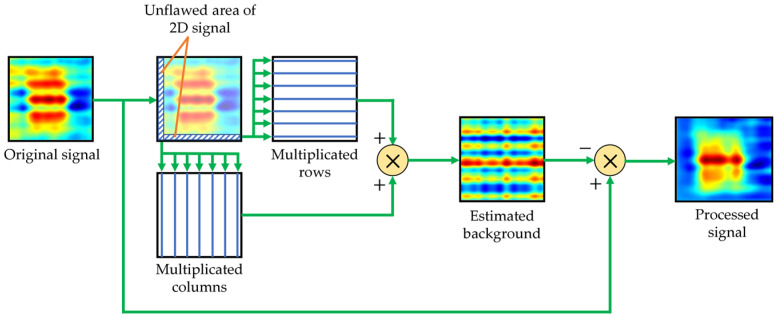
Signal processing algorithm for the background signal removal.

Figure 9 shows the relative voltage difference ∆u as a function of the transducer’s position along the *x*-axis and *y*-axis. The flaws OF70% (Figure 10a) and OF30% (Figure 10b) are detectable, but a high material anisotropy caused by the fiber alignment affects the results. Especially for minor hidden flaws, this makes it more difficult to detect and localize them. The undoubted advantage of the presented transducer, sensitivity to defects of different orientations in the case of anisotropic materials such as carbon fiber-reinforced composites, generates interfering signals that make analysis more complicated. The problem can be overcome by changing the design of the transducer or by using image processing algorithms involving background removal. This study proposed a dedicated background removal algorithm (Figure 11).

**Figure 12:**
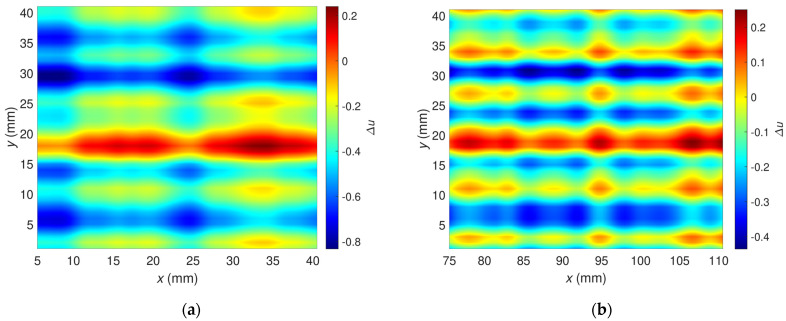
Two-dimensional plots of the background signals estimated for the excitation frequency *f* = 2 MHz, which were utilized to correct signals measured for the flaw: (**a**) OF70%, (**b**) OF30%.

The algorithm block scheme is shown in Figure 11. Rows and columns from the signal’s data matrix, corresponding to the unflawed part of the sample, are selected and multiplicated. On their basis, the signal background is estimated. The procedure ends with subtracting the estimated background signal from the measured signal. Examples of the signal background estimated for OF70% and OF30% are depicted in Figure 12.

**Figure 13:**
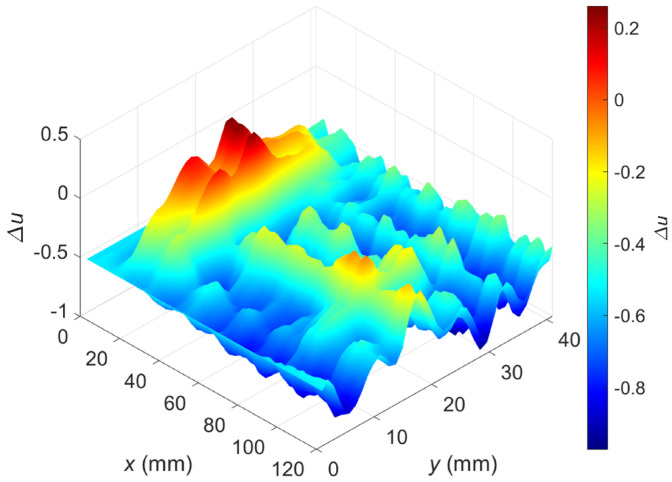
Two-dimensional plot of the relative voltage after background removal; excitation frequency *f* = 2 MHz.

Figure 13 shows a two-dimensional plot of the measured relative voltage after background signal removal (the whole measurement area with both flaws was included). Compared to the signal before processing (Figure 9), one can observe that the interferences caused by the heterogeneity of the carbon fiber-reinforced composite were minimized and both flaws are detectable.

**Figure 14:**
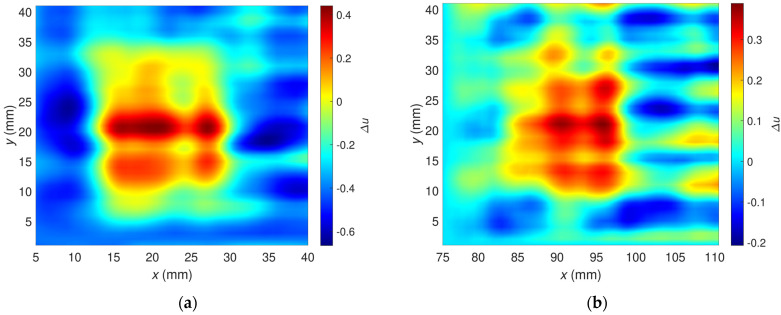
Two-dimensional plot of the relative voltage after background signal removal; excitation frequency *f* = 2 MHz; plot for the flaw: (**a**) OF70%, (**b**) OF30%.

Figure 14 was prepared to focus only on the sample area around the flaws. It illustrates the relative voltage ∆u as a function of the transducer’s position along the *x*-axis and *y*-axis after applying the background removal algorithm. Compared with the non-processed signal (Figure 10), the flaws OF70% and OF30% can be detected considerably better here. Removing the background signal caused by the material anisotropy diminished the analysis difficulty and minimized the hazard of incorrect flaw classification.

**Figure 15:**
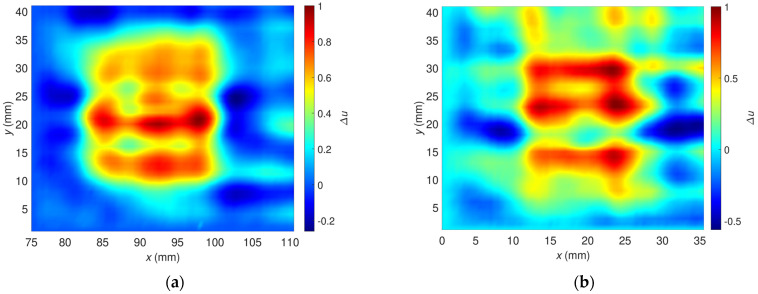
Two-dimensional plot of the relative voltage after background signal removal; excitation frequency *f* = 2 MHz; plots of the signals measured for the same side flaws (inner flaws): (**a**) IF70%, (**b**) IF30%.

The measurements conducted for the outer flaws confirmed the usability of the proposed system. A relatively low excitation frequency (2 MHz) was used to detect hidden defects (inner flaws), which guaranteed a sufficiently large penetration depth and the ability to detect even shallow defects. Unfortunately, such an excitation frequency is not optimal for detecting surface defects because the eddy currents should be concentrated in the near-surface layer. Therefore, additional measurements were performed for surface defects to confirm the system’s effectiveness in this case as well. Figure 15 shows plots of the relative voltage *∆u* as a function of the transducer’s position along the *x*-axis and *y*-axis. In the case of this measurement, the defect names were changed respectively: OF70% becomes IF70% (Figure 15a), while OF30% becomes IF30% (Figure 15b). It is evident that both defects are easily detectable. These results confirm that the transducer enables the detection of inner and outer flaws using the same excitation frequency.

## 4. Conclusions

Currently, the eddy current technique is widely used to inspect metal parts. There are many ongoing intensive works to adopt existing ECT systems to evaluate composite structures consisting of conducting materials such as carbon fibers. In the described studies, intensive work was carried out to adapt a well-proven (in the case of metals) system and transducer. Particular emphasis has been placed on providing the ability to detect hidden defects (inner flaws).

The conducted experiments are generally promising. However, the method requires further development and adjustment to the specificity of the composites. Experiments carried out so far have allowed several conclusions to be drawn:

- A differential transducer allows for a significant increase in sensitivity, but placing the reference sensor close to the measured material is not an effective solution due to the anisotropy of the tested material; a more effective solution seems to be the use of a reference sensor with the reference material;
- The ability to detect defects can be improved by using signal processing, such as the proposed background signal removal;
- The use of resonant frequencies allows for multiple increases in the sensitivity of the transducer; at the same time, it is more complicated to use the frequency response to identify the type and depth of the defect;
- The use of even a relatively low excitation frequency that guarantees the appropriate depth of penetration does not prevent the effective detection of surface defects;
- The use of the frequency response to defects identification in the case of the composite materials requires the use of a much wider range of excitation frequencies.

The experiment was limited to detecting notches (corresponding in some way to cracks) to demonstrate the system’s usability. Despite satisfactory results, a comprehensive study on detecting other flaws, such as delamination or porosity, should be conducted to ensure the method’s versatility. Moreover, additional research on the method’s applicability is needed for structures with different internal reinforcement weights and resin-rich samples.

## Figures and Tables

**Table 1:** Sample parameters.

Sample S03	
Dimensions	210 mm × 148 mm
Thickness	2 mm
Mass	ca. 90 g
Face reinforcement weight	$245\frac{g}{m^{2}}$
Internal reinforcement weight	$400\frac{g}{m^{2}}$ and/or $600\frac{g}{m^{2}}$
Surface coating	matt coating
Heat treatment	annealing at a temperature of 60 °C

**Table 2:** Transducer parameters.

Parameter	Value
Excitation coil *E_A_* turns	25
Excitation coil *E_B_* turns	25
Excitation coil *E_C_* turns	25
Excitation coil *E_D_* turns	25
Pick-up coil *S* turns	100
Ferrite core diameter	12 mm
Excitation coils—pick-up coil distance	5 mm
Core column diameter	2 mm
Core column height	6 mm
Core height	10 mm

## Data Availability

Not applicable.
